# The Stacked Community Engagement model: A practical model for developing community-engaged academic medical faculty

**DOI:** 10.1017/cts.2023.1

**Published:** 2023-01-11

**Authors:** Bryan Johnston, Leslie Ruffalo, David Nelson, Sarah O’Connor, Erika Petterson, Staci Young

**Affiliations:** 1 Department of Family and Community Medicine, Medical College of Wisconsin, Milwaukee, WI, USA; 2 Office of Community Engagement, Medical College of Wisconsin, Milwaukee, WI, USA

**Keywords:** Community engagement, community-engaged research, translational research, faculty development, academic medicine, tenure and promotion

## Abstract

**Introduction:**

There is an increasing recognition of the benefits of sustained community engagement (CE) that accrue to academic health centers and the communities they serve. However, the success and sustainability of CE projects rely on the efforts of individual faculty, learners, and community members, for whom CE efforts are typically added to their professional and personal priorities and responsibilities. This competition for time and resources between priorities and CE can discourage academic medical faculty from participating in CE activities. The Stacked Community Engagement model is proposed to synergize or “stack” responsibilities and goals onto the scaffolding of CE projects.

**Methods:**

We examined the literature and expert CE practitioner opinion to identify the challenges faced by community-engaged academic faculty and the key characteristics of CE projects that successfully align and integrate with the priorities of faculty, learners, and community members. We synthesized this information to develop the conceptual Stacked CE model for developing CE academic medical faculty, then illustrated the model in heterogeneous CE programs to explore its generalizability, validity, and robustness.

**Results:**

The Stacked CE model, when applied to a specific nutrition education program (The Food Doctors) and outreach program (StreetLife Communities), provided a practical framework for examining the sustained success of a partnership between Medical College of Wisconsin faculty and medical students and the community.

**Conclusions:**

The Stacked CE model is a meaningful framework for developing community-engaged academic medical faculty. By identifying overlap and integrating CE into professional activities with intention, CE practitioners can benefit from the deeper connections and sustainability.

## Introduction

Academic medical faculty lack a practical model for developing and integrating community engagement (CE) skills and responsibilities into a meaningful and sustainable career trajectory. Without a model that focuses on leveraging the organizing potential of CE to align faculty efforts and goals, CE often seems “extra” or “additional,” an inefficient method for developing an academic medical career. As such, CE often languishes in favor of other more traditional areas of accountability and expectation such as research, teaching, and clinical care. This is unfortunate, as CE has the potential to help academic medical faculty reach many—if not all—internal and external goals and unify their efforts by “stacking” goals and expectations into the space of a single project. In activating the potential of CE in this manner, academic medical faculty have an opportunity to focus on fewer areas, exploring each more deeply, meaningfully, efficiently, and sustainably. Toward this end, the Stacked CE model is described below and illustrated through application to real-world projects. It is hoped that uptake of this model will enable academic medical faculty passionate about CE to craft more meaningful careers better aligned with their passions, that more learners will engage in CE with the “stacked” perspective, and that communities will ultimately benefit from more effective CE.

## Background

**Fig. 1.**
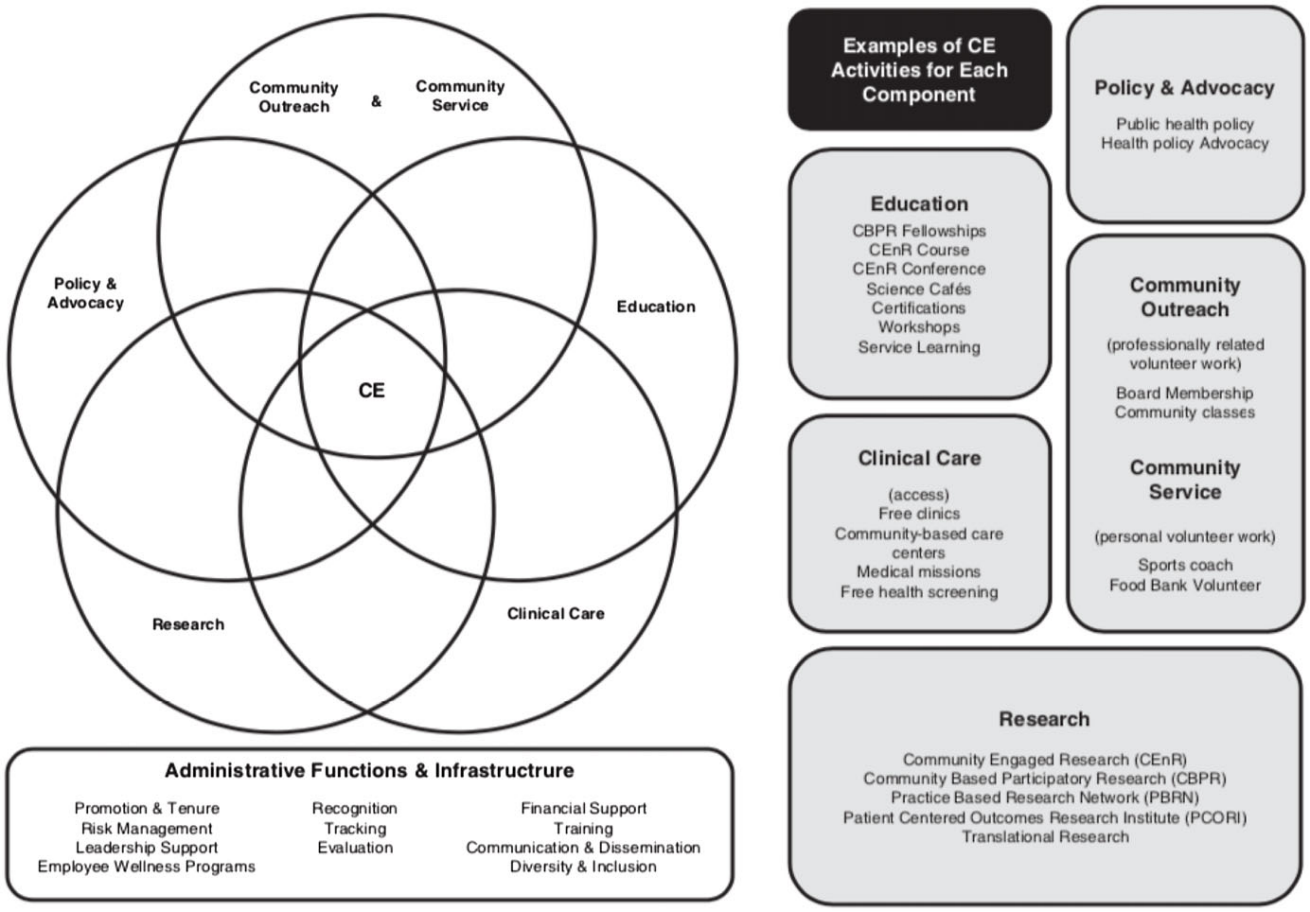
The community engagement (CE) Components practical model of Ahmed and colleagues [7].

Recognition of the critical role of CE is growing within the academic medical community [1-4]. The value CE represents for academic health centers (AHCs) has become more evident, including their increased ability to impact the health of the communities they serve, their potential to develop productive partnerships and build capacity [3,5], the framework they provide for the pursuit of scholarly work and grant funding [1,5,6], and their potential to drive health equity [4]. As institutions move toward embracing CE principles and methodologies, institutional models for embracing and promoting CE have been developed and disseminated. The CE Components practical model of Ahmed, Neu Young, DeFino, Franco, and Nelson [7], which describes the many dimensions of CE—including community outreach and community service, education, clinical care, research, and policy and advocacy—alludes to areas of intersection between those dimensions (Fig. 1). Many curricular approaches have emerged to communicate CE concepts to AHC faculty and staff—including virtual synchronous and asynchronous programming [5,8], longitudinal coursework [9], textbooks [10] and other guided curricula, and cohort-based training [11]. These educational interventions, broad dissemination, and advocacy efforts represent the potential for transformation in CE conceptual understanding within AHCs [12].

**Fig. 2.**
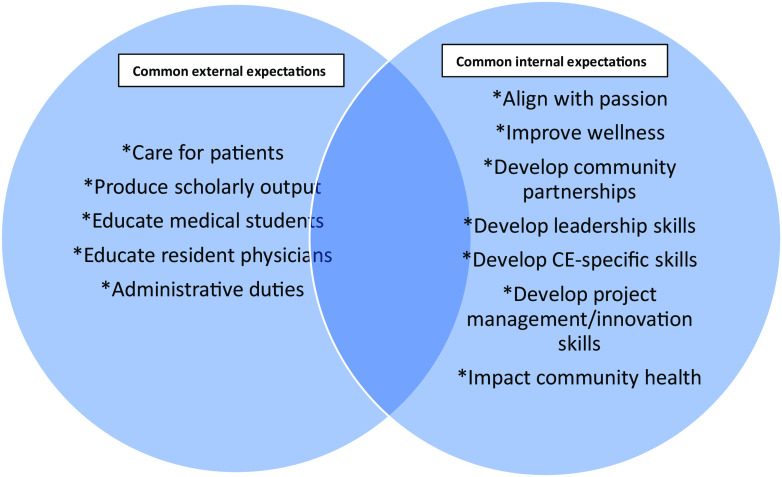
Common academic faculty priorities.

Historically, less focus has been dedicated to supporting academic medical faculty to develop capacity for learning and applying CE skills and methodologies [13]. These individuals commonly face additional institutional demands and expectations in many domains (Fig. 2) and must balance that with their internal motivations and priorities. Critically, although CE may be supported or encouraged at the institutional level, it is rarely elevated to the level of importance of other roles and expectations that a faculty member may be subject to in their career [14]. There may be an enthusiasm for the importance of CE, but there also may be a sense of CE as professionally risky [1] or in competition with traditional faculty activities [15].

A perceived lack of time puts CE in direct competition with internal and external expectations and priorities for many faculty. Chung and colleagues [16] surveyed the UCLA Clinical and Translational Science Institute’s faculty about barriers to conducting CE research. Faculty pointed most frequently to a lack of time (73.6%), but they were also concerned about the lack of (1) funding opportunities (58.5%), (2) access to community partners (27.9%), (3) incentive/reward structure (23.1%), and (4) capacity/skill (21.4%).

As a result of this perceived lack of time and support, other priorities often edge out CE. For example, faculty members may choose between dedicating time to clinical or scholarly activities and CE work. They may de-emphasize CE work due to a more direct perceived link between these other activities and financial or promotion incentives. In a professional field in which time is a continual limitation, departments may dissuade CE-interested faculty from developing capacity for CE or reaching their potential as CE practitioners.

**Fig. 3.**
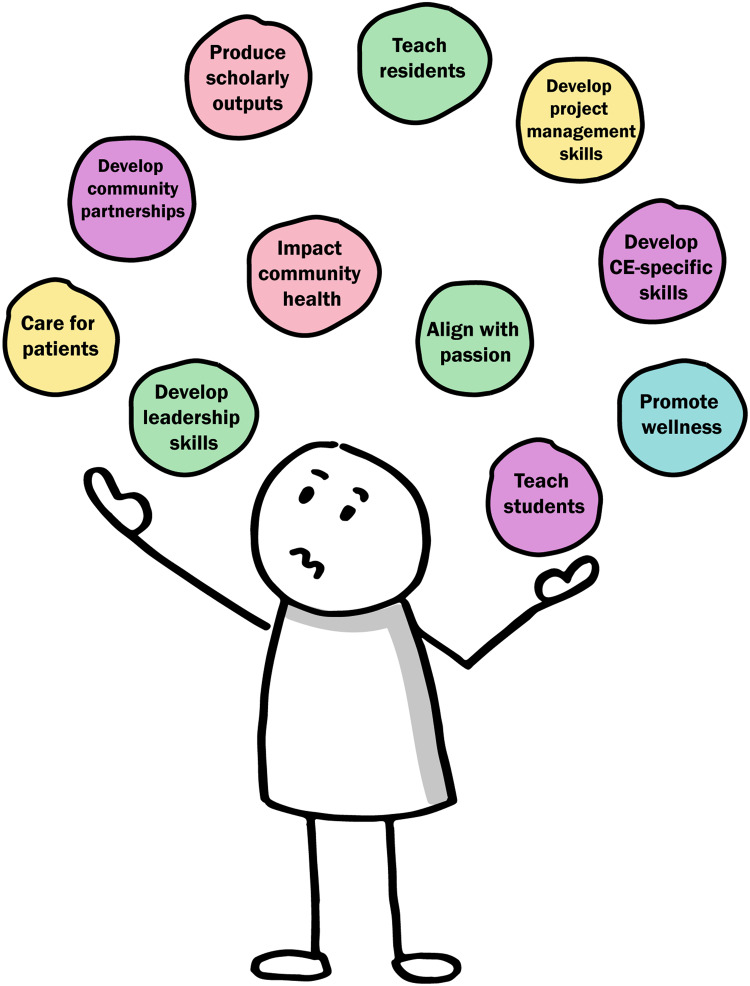
The “many hats” model of academic medical faculty organization can make community engagement feel like another thing to juggle.

Not supporting CE may be the result to some extent of the “many hats” approach that has gained traction as a method to facilitate faculty role switching [17,18]. In the “many hats” approach, faculty progress through one task at a time in a linear, additive fashion, pausing to apply a different tactic or behavior for each new task in which they are engaged (Fig. 3).

The “many hats” approach promotes dividing and isolating tasks, and the more intersectional aspects of the CE Components practical model can be difficult for faculty to reconcile. Without access to mentors in CE or opportunities to gain valuable experience applying CE in an intersectional manner, this seeming discrepancy can make it difficult in practice for faculty to actualize their intention to develop as CE practitioners. Academic medical faculty pursuing CE through the traditional or “many hats” approach may perceive CE goals as added in succession to their many other goals, along with an additional required expenditure of effort. The frequent task-shifting demanded by this approach can cause feelings of juggling, reactivity, inefficiency, and being overwhelmed.

No matter how adept a faculty member may become at juggling discrete tasks, they will continue to struggle with the innate competition of expanding activities for finite time. This time constraint will not change, and frustration and burnout are the predictable outcomes of a situation where CE is only added to traditional faculty activities rather than integrated into them [19].

Leaving this discrepancy unresolved would be unfortunate. Similar to the findings of Chung and colleagues at UCLA in 2015 [16], a 2018 analysis of a Medical College of Wisconsin (MCW) institution-wide CE survey identified time limitations as a common barrier to faculty CE involvement [20]. Experienced faculty find this a common challenge in CE. Bloomgarden and O’Meara [15] surveyed perspectives of CE among faculty moderately or highly involved in CE and found that 15% of these active CE faculty respondents compartmentalized their CE activities, and 60% only sometimes found their CE harmonized with their traditional academic activities or could benefit from integration. Only 25% had adopted an integrated view of CE in their wider professional activities. Even experienced CE practitioners may benefit from engagement in approaches to integrate CE more thoroughly into their professional roles. To that end, we have developed a conceptual model, the Stacked CE model, to demonstrate the capacity of CE to align academic medical faculty efforts. Here, we describe the model from the perspective of faculty and illustrate its application.

## Priming the Model

We identified several key factors that enable the successful application of this model. These factors are critical in creating an environment conducive to successfully unlocking the potential of the Stacked CE model. If these factors are not present in an academic or community setting, it may be necessary to bring focus to these areas alongside or prior to application of the model.

### Robust Community of Support

Community Engagement largely exists in the community, and thus relies on robust community factors, including effective and engaged community partners, organizers, local political and community leaders, and participation of the public in general. Academic medical centers must support community interests and in doing so cultivate reciprocal trust and support from the community. Local circumstances differ significantly in terms of community health challenges and priorities, community organization and leadership structure, local political and social dynamics, and historical and present relationships with AHCs. Without a robust opportunity to support the community, the Stacked CE model—and robust CE in general—may not yet be suitable to apply in its full extent.

### Longitudinality

CE diverges from traditional healthcare educational structures by requiring a longitudinal commitment that does not neatly fit into the time frame of a single rotation or course schedule or the weekly barrage of tasks and requests that academic medical faculty navigate [21]. Community-engaged relationships and processes move on an incremental pace as a rule—conversations at an introductory level, often without tangible agendas or outcomes, are often necessary at the initiation of community–academic relationships, and effective and sustainable project and research design similarly involve longitudinality [22,23]. It is advisable to identify and leverage longitudinal engagement opportunities, including longitudinal learner and faculty tracks, flexible and supportive learner and faculty expectations and scheduling, and attention to sustainability from the start to account for the potential turnover on all sides of the partnership. In the context of the Stacked CE Model, it takes time and focus to collaboratively create a structure achieving the intricate and dynamic level of alignment promised.

### Training and Mentorship

Who is currently doing CE work at a high level? Who has navigated the systems to enable CE efforts to reach their stacked potential? Is there a readily available answer when CE-interested faculty, learners, or community members ask, “Who can help me with this?” Identify those individuals and teams and activate them as supports and mentors to those seeking CE work. Individuals with highly developed CE and mentoring skills can provide support in building CE capacity, but they can also connect people and resources, amplifying individual and collective efforts. In the Stacked CE Model, this may represent an advisor or supporter who from their different perspective and experience can visualize the whole of the project and the advise appropriate placement of pieces.

### Institutional Support

Institutional support is critically important in promoting sustainable faculty involvement and development as CE practitioners. Sustained CE programs play a crucial role in building and maintaining trust in the community [24]. The higher the level of and the more action-oriented the institutional support, the more cover it provides for faculty and institutions to engage in, advance, and succeed at CE. Adopting CE as part of the institutional mission and vision fosters an environment in which CE can thrive across the institution [6,25-29] and one in which CE efforts, including community-engaged research (CEnR), teaching, and clinical work, are considered part of the academic enterprise [1,6,29] and tenure and promotion process [30]. Conversely, frustration with a perceived lack of understanding of CEnR as a “valid” approach to medical and public health research, coupled with a lack of pilot funding and protected time, can isolate CE activities, putting them in direct competition for time [19] and leading to faculty de-emphasizing CE.

Establishing an institutional infrastructure that supports CE provides faculty with the resources necessary to build the capacity needed to sustain meaningful CE [1,13]. This infrastructure can take many forms, including recognition of CE efforts, provision of protected resources like time [13,16,19,31] and funding [13,16,19,28,32] to CE efforts, continuing education, and recognition of CE as salient to the review, promotion, and tenure process [6,14,16,23,28,32-34]. Programs and models are developed into the space provided and rewarded by their environment. In the context of the Stacked CE model, the space environment and respect for this type of effort are critical factors to enable its application.

### Acknowledgement of Historical Context

Healthcare and academic institutions have not always historically been good partners to the communities they serve, in some cases “poisoning the well” for future potential CE practitioners [13]. This may be particularly true for underserved communities and communities of color, which may have many reasons for distrust of medical researchers [35,36]. The trauma and sequelae of such violations of trust can last generations [37]. It is important to identify and acknowledge the academic medical center’s historical and current reputation in the community [18] when pursuing true CEnR and, if necessary, seek to reconcile or account for this standing before proposing new projects and partnerships [24]. Bidirectional communication, a crucial CE skill, can aid the CE practitioner in navigating the disambiguation of perceived reluctance in a prospective community or individual partner. This can have a powerful impact on community participation. For instance, Wendler *et al*. [38] found that, when barriers to participation are acknowledged and accounted for, Black and Hispanic individuals participate in health research at higher rates than non-Hispanic White individuals. Alongside institutional efforts to build trust and increase access to the health resources the institution can bring to bear, individual CE practitioners must be cognizant to build trust on an individual level—for instance, engaging without an agenda, starting with questions and not with answers, demonstrating accountability and follow-through, and, when appropriate, acknowledging historical and current barriers to trust and participation. The Stacked CE Model requires collaboration with community and learner stakeholders and relies upon the capacity for all sides to participate in trusting relationships with one another towards mutual goals.

## The Stacked CE Model

**Fig. 4.**
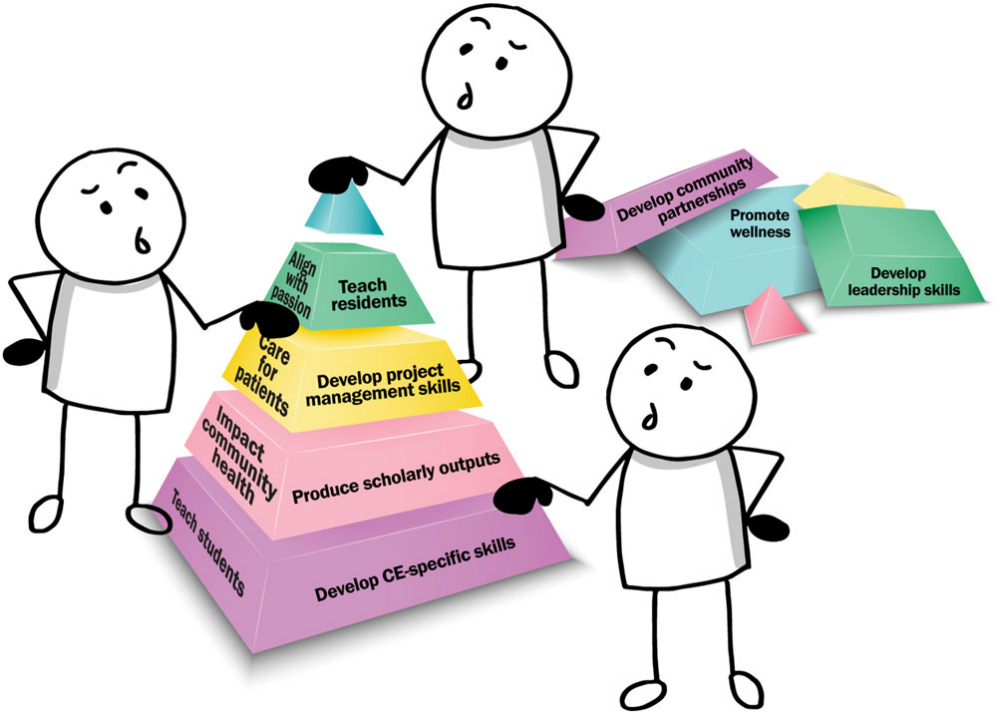
The Stacked community engagement model.

The Stacked CE model in Fig. 4—representing the perspective of an academic medical faculty member approaching CE in collaboration with others, such as community partners and learners—illustrates the potential of CE to align and harmonize faculty efforts. The model is constructed in a familiar block-stacking format and includes blocks representing internal goals and external responsibilities common to academic medical faculty. Similar to a game of Tetris®, if the blocks are allowed to just fall without intervention, they quickly stack up and the game is lost. This simple additive approach stacks the responsibilities high and wide and leaves a lot of gaps. But when the blocks are carefully arranged, the height of the wall is managed and the gaps are few, if any. This careful arrangement allows the faculty member—in collaboration with community and learner partners who may also be approaching the work from a stacking perspective of their own—through creativity, perspective, and dynamic alignment, to unify and “stack” responsibilities and goals in a more manageable and accessible format that facilitates synergistic connections between them all.

The fundamentally holistic CE approach and the types of activities possible in the breadth of CE act as levers to amplify effort and reach many varied goals at once. In a longitudinal CE project, it is reasonable to possess common faculty goals and responsibilities. Independent and more episodic CE projects that may not always extend to these other areas—for instance, isolated service projects such as a food drive—may also carry the potential to do so. Leveraging a food donation service project to include student or resident learners as community-engaged learning participants may consist of opportunities to build connections with community partners and resources, may involve a scholarly approach to evaluating program process and impact, and may contain opportunities to connect with clinical efforts such as behavioral counseling or food security screening and intervention (which itself may represent scholarship opportunities). By identifying this potential intersection, a faculty member involved in such a project may now have the opportunity to synergize the activity and meet education, scholarship, leadership, and skill development goals all in one project. They may choose to shift focus away from other projects which might have met these goals and responsibilities but represent a path with less efficiency or passion. CE nearly always has the potential to act as a stacking or aligning force for faculty effort.

It is important to note that each faculty member will have different goals, responsibilities, and priorities. It is recommended that when preparing to utilize the Stacked CE model, faculty spend time examining job descriptions, tenure and promotion guidelines, and their own individual personal and professional goals. It may be advisable to discuss these factors with mentors and/or leaders for assistance in determining where alignment may be possible. As careers and projects progress, these factors may also change, requiring revision of general internal and external priorities. Not all plans and responsibilities will carry the same weight, either—the model is not to scale. The model symbolizes circumstances commonly faced by academic medical faculty pursuing CE. The model illustrates that, with lower relative effort, faculty engaged in CE can address many goals and expectations in a manageable and even enjoyable manner.

To date, the model has been applied in flexible formats – as a conceptual presentation for faculty, learners, and community members, as a prospective planning tool as projects are being conceptualized or initiated, and as a tool to challenge and expand thinking on already-active CE projects which may represent opportunities to grow in depth and reach. The model has been presented in the context of example projects and used to stimulate individual reflection, group discussion, and to provide framework for mentorship. Future efforts or applications of the model could include structured opportunities for CE stakeholders to identify their own personal internal and external priorities, either as an individual exercise or in partnership between academic, community, and/or learner partners. Rigorous application of the model could take the form of workshops for already-established partners and matchmaking opportunities for those interested in new collaborations, with potential integration into project timelines as a periodic check-in exercise as projects develop, to ensure all involved parties are optimizing their impact and alignment with their priorities at each stage of the project.

### Application of the Model: The Food Doctors

The Stacked CE model is illustrated through its application in the case of a community-based nutrition education program called The Food Doctors (TFD) [39]. The project was initiated by a partnership between medical students passionate about teaching and nutrition and a community K-12 school that had identified nutrition education as a curricular need. With the backing from their CE-skilled faculty advisor, the medical students collaborated with leaders of the partner school to develop innovative curricula designed for this specific student population. Initially the primary purpose—from the perspective of the medical students and school leaders—was to provide high-quality nutrition education to elementary students who lacked that opportunity. Over time with mentorship of the CE-skilled faculty advisor, the students were encouraged to apply CE-specific skills, to design and conduct program evaluations, to disseminate in both community partner and academic medical settings, and to create sustainability procedures.

Over the course of several years, the project evolved from a flat and relatively simple project to a stacked project representing many areas of internal and external value for medical students, school leaders, and CE-skilled faculty. For instance, the extension of the project from a simple service activity to a dynamic CEnR partnership with a scholarly foundation and financial and team-based sustainability planning enabled significant additional benefits to the CE-skilled faculty, the school partners, and the medical students themselves. Over the course of a decade, the CE-skilled faculty member supported generations of medical students and school leaders in joining the stacked program, maintaining and growing the program as representatives of each side have moved on to other roles or institutions. Each new generation of students and school leaders has had the opportunity to engage by learning in CEnR, leadership, and project management skill development. Scholarly questions have emerged from both the community and academic sides of the partnership, and as scholarly directions have been pursued, lessons learned have been shared with all partners to inform understanding and practices on the community, medical learner, and academic faculty sides. Each student leader has launched a unique area of scholarship related to the project and its impact on participants, families, teachers, and other related aspects of community health. The project has bridged the clinical setting by expanding nutrition education opportunities for an underserved primary care clinic population, assessing for and intervening upon food insecurity, and expanding education efforts to include resident physicians and faculty.

The environment primed the TFD program for success (i.e., the longitudinality, community partnerships, training and mentorship, institutional support, and historical acknowledgement factors were in place), and TFD has been successful by multiple measures: robust scholarly and community-level dissemination has emerged from this project, creating dynamic gains in knowledge, skill development, and career development [39,40], and MCW conferred its highest institutional award for CE on TFD. Generations of elementary students have experienced high-quality nutrition education and access to medical students skilled in CE who, in turn, have gained numerous skills, experiences, and perceptions of synergy between personal and professional priorities to propel them toward effectiveness and satisfaction in their careers.

In a sense, this project, started as a direct service activity, saw its potential unlocked by a longitudinal stacking process developed over the course of several years of mentorship from a CE-skilled faculty member. The faculty member challenged student participants to stack the program gradually over the course of years, and subsequent generations of learners and community partners have been able to join in and benefit from the project in its stacked form.

**Fig. 5.**
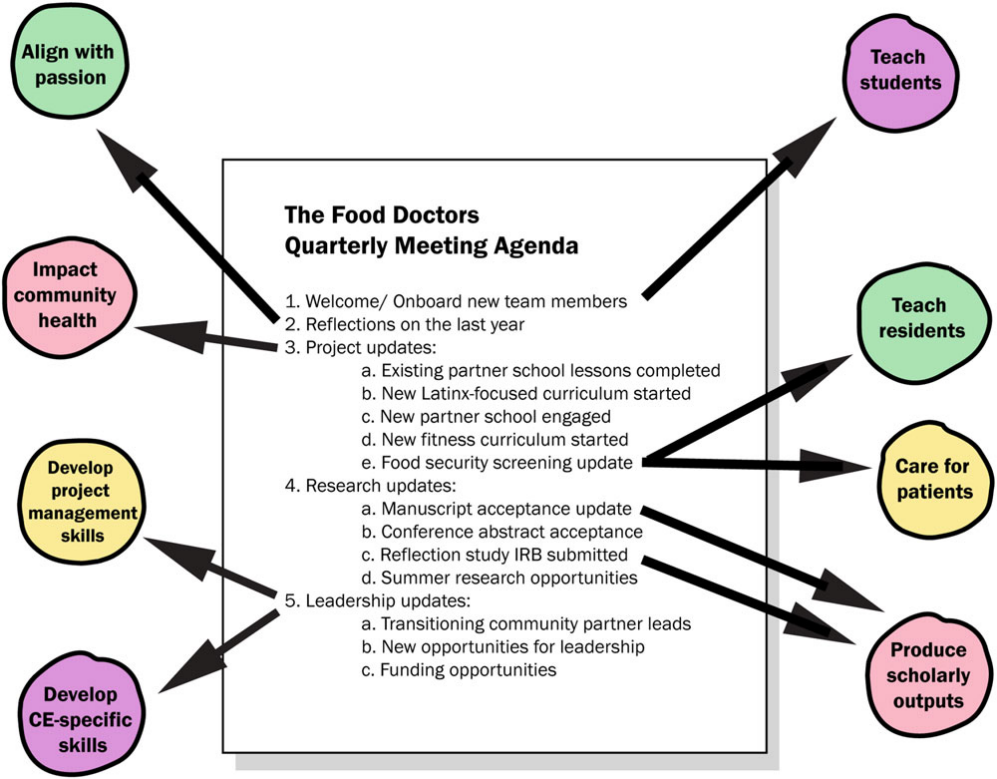
A recent The Food Doctors team meeting agenda illustrates how a project in which the Stacked community engagement (CE) model is applied fulfills common academic faculty priorities.

From the faculty perspective, this project touches on all common academic faculty priority areas from the Stacked CE model. Mapping the agenda from a recent TFD quarterly project team meeting demonstrates how the faculty-centric model is presently applied and reaches diverse faculty priority areas (Fig. 5). The agenda for this hour-long meeting, developed by student leaders with faculty input, brings together myriad intersecting aspects of this CE project, and represents the manner in which this project has continued to remain stacked.

From the perspective of learners and other project stakeholders, this inclusive approach to project management ensures project sustainability independent of individual faculty, a key characteristic of successful CE that the program’s decade-long partnership has demonstrated. In this example, although an urgent patient care issue detained the faculty director, the meeting continued in the hands of capable medical student leaders. This kind of intentional stacking of impact has significant implications for allowing medical learners and faculty to engage in multiple areas of priority efficiently and effectively.

### Application of the Model: StreetLife Communities

Another illustration of the Stacked CE model is in the area of housing security. One of the authors (DN) first started volunteering at a food pantry, which evolved into an initiative, StreetLife Communities, to conduct street-level outreach to the unsheltered homeless population, many of whom were served by the pantry but had various barriers preventing regular pantry access. Although the original intent was not to do scholarship, engage learners or other goals besides direct service, the development of scholarship supports telling the narrative around the topics like homelessness and the impact on the community [41]. In turn, this resulted in the engagement of other students and faculty and their inclusion in the scholarly effort [42]. In this way, the project grew over time from a direct service activity into a stacked CE project. The changes above were arrived at in a pragmatic stepwise fashion, but once in place, the introduction of learners, additional faculty, and scholarly work into the strong existing community-academic partnership created an environment in which stacking activities rapidly developed for all involved.

The project has progressed as academic medical faculty and learners, community leaders, and community members have evolved their relationships and roles over time, often in overlapping ways. Deep commitment to the community and multiple regular contacts with unsheltered community members each week enmeshed medical faculty and community partners in the dynamics of the unsheltered homeless community, and they became known as trusted supporters. As some currently or formerly unsheltered community members gained familiarity and developed positive relationships with the program, some chose to join the outreach effort in both compensated and volunteer roles. After a period focused on colearning and relationship development, medical learners and faculty were introduced to the community and engaged in a stepwise fashion, eventually conducting research and collaborating on clinical projects which were identified as priorities by the community and community partners.

From the faculty perspective, this project illustrates the strength of many facets of the stacked CE model. Initially conceptualized as a mechanism to partner with community leaders around an area of passion to improve the health of vulnerable community members, faculty have benefited from the application and development of CE-specific skills and from the tangible and intangible benefits of developing a strong community partnership. As the program and relationships within it have matured, faculty have incorporated external expectations like medical education and scholarly output, and the inclusion of clinical faculty and faculty interested in street medicine practices has generated significant momentum toward providing robust street-based medical care in a community of high need. Finally, widening the scope of influence by enabling new academic medical leaders to develop in this area has allowed the initiating faculty member to slow the pace of direct outreach involvement and focus on advocacy, writing, and other evinstance, learners and community olving career and wellness needs, many of which also meet community-level and learner-level needs in new and different ways.

Learners, community partners, and community members have similarly benefited from the stacked components this project has come to exemplify. Community partners have found their capacity enhanced, and the demonstration of their value reinforced by what the learners and scholarly outputs added to the program. Community partners have developed a better understanding of how to engage with the healthcare system and have appreciated the opportunity to influence and educate the next generation of physicians. Areas of inquiry including the health needs assessment have improved community partners’ understanding of how to prioritize initiatives to support the community. Stronger connections with physicians and the healthcare system have enabled more timely and impactful support to community members with health concerns. Community partners have also benefited from having a voice in the initiation of street-based healthcare initiatives.

## Discussion

The CE Stacking model provides a promising approach to inspire mindset shifting for faculty seeking to connect their interest in CE with the disparate goals for learning and career development commonly ascribed to their roles. With this model, we have created both a message about the benefits CE can bring to academic medical faculty and institutions and their communities and a tool, the Stacked CE model, for finding the synergy that can result from integrating CE with the traditional activities of academic medical centers—teaching, scholarship, and clinical work.

The capacity to enact more impact in the same allotted time confers resilience to the various projects in which faculty may be involved. This resilience is crucial to the sustainability of CE projects by a collaboration of faculty, students, and community partners at a level independent of the participation of any specific individual—an important goal of rigorous CE. Absent that pathway or vision, stakeholders interested in CE may find their projects never achieve multidimensionality and diverse impact, two characteristics that perhaps best represent the potential CE holds. Instead, they may find their projects occupy one role or one goal and simply add another linear task or responsibility to their already busy schedule—one that takes yet another hat and another unit of focus and effort. Without alignment with other more objective internal goals or external responsibilities, such an activity would be vulnerable to falling away when capacity is exceeded and the hard decisions about what activities will continue to be pursued. What is more, lacking perspective or awareness of CE’s stacking potential and the momentum it can confer upon CE activities, CE-interested stakeholders may find themselves trapped in an inert state and never reach the point at which their interest in CE tips into actual CE activity.

Faculty new to CE, especially those familiar with or even proficient in the additive and linear “many hats” approach, may need to develop their CE skills and projects before they reach the point at which there is the critical mass required for stacking potential. These potential Stacked CE practitioners may require stepwise support to move towards a stacking approach, similar to the measured approach to CE espoused in the Steps model [22] in which the practitioner of the Stacking model may progress through stages of its application, from the conceptual stage, to the educational stage, to the motivational stage. CE-skilled mentors are key in supporting this evolutionary process.

The CE Stacking model may be flexibly applied and thus has the potential for wide resonance. To date, the model has been applied in the context of example projects in group formats to stimulate discussion and reflection about current and future projects. It has been applied as a tool in individual mentorship relationships and project team management by CE-skilled practitioners as a way to inspire stacking approaches and challenge “many hats” approaches. Further applications may include building additional structures to support individual and collective definitions of internal/external priorities and longitudinal processes to maximize the stacking nature of CE projects.

The CE Stacking model has the potential for application to other stakeholders engaging in CE—for instance, learners and community partners [43-46]. These groups are expected to have some shared internal goals and external expectations compared with academic medical faculty—including engaging in areas of passion, producing scholarship, developing leadership and CE skills, pursuing funding, and impacting community health. There may be some areas of divergence—for instance; medical learners may also prioritize personal and professional identity formation, mentorship, research skill development, learning about and impacting social determinants of health, and developing health communication skills. Community partners may prioritize access to health care resources, expertise, and relationships. Of course, individual faculty, medical learners, and community partners are expected to demonstrate individualized goal and expectation lists and prioritize their lists differently. However, there does seem to be an opportunity for the applicability of this model to the situations of the major stakeholders involved in CE. Perhaps more importantly, synergizing the application of this model between the three groups, perhaps in planning stages of a new initiative or at intervals as a project develops, could have the potential to improve transparency and collective benefit of all stakeholders of CE projects.

This conceptual model emerges from CE faculty expert reflection on best practices, application to selected cases, and feedback from application in education and mentoring initiatives. The model provides a theoretical framework to promote and foster CE activity among academic medical faculty. Still, other applications could include education in many formats, mentorship, professional development, and faculty development. The intersection between faculty perspectives and that of community partners and learners is another area of significant potential. Further inquiry may consist of an evaluation of the model’s impact and further exploration of its validity and robustness.
